# Mechanical and Dynamic Behavior of an Elastic Rubber Layer with Recycled Styrene-Butadiene Rubber Granules

**DOI:** 10.3390/polym12123022

**Published:** 2020-12-17

**Authors:** Seongdo Kim, Hyun-Oh Shin, Doo-Yeol Yoo

**Affiliations:** 1 Department of Agricultural and Rural Engineering, Chungnam National University, 99 Daehak-ro, Yuseong-gu, Daejeon 34134, Korea; sdpcid@kcl.re.kr; 2 Department of Architectural Engineering, Hanyang University, 222 Wangsimni-ro, Seongdong-gu, Seoul 04763, Korea; dyyoo@hanyang.ac.kr

**Keywords:** synthetic sports surface, elastic rubber layer, styrene-butadiene rubber, tensile properties, shock absorption, vertical deformation

## Abstract

This study evaluates the tensile properties, including the tensile strength and elongation at break, and dynamic behavior, including shock absorption and vertical deformation, of an elastic rubber layer in synthetic sports surfaces produced using waste tire chips containing styrene-butadiene rubber (SBR). The primary variables of the investigation were the number of compactions, resin–rubber granule ratio, and curing conditions, such as aging, the temperature, and the relative humidity. The test results showed an increase in the tensile strength of the elastic rubber layer with recycled SBR as the number of compactions, resin–rubber granule ratio, curing period, and temperature increased, while the elongation at break was affected by the curing temperature and period. Shock absorption and vertical deformation decreased with an increasing resin–rubber granule ratio and number of compactions due to the increased hardness. However, these properties were not significantly affected by the curing conditions. Furthermore, the test results indicated that the curing temperature has a pronounced effect on the tensile properties of the elastic rubber layer, and maintaining the appropriate curing temperature—approximately 50 °C—is a possible solution for improving the relatively low tensile properties of the elastic rubber layer.

## 1. Introduction

**Figure 1:**
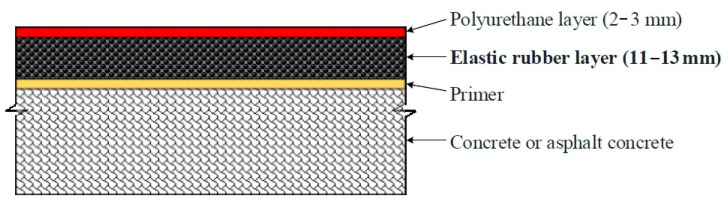
Structure of a synthetic sports surface.

Synthetic sports surfaces for outdoor sports facilities, such as athletics, track, field, tennis, and multi-sports, are widespread. In general, synthetic sports surfaces are installed on concrete or asphalt concrete bases and comprise a 2–3 mm-thick polyurethane layer and an 11–13 mm-thick elastic rubber layer, as shown in Figure 1. The elastic rubber layer is the most important part of a synthetic sports surface because it constitutes the largest portion of the surface and reduces impact forces when athletes make contact with the surface. This layer is usually produced by mixing recyclable synthetic rubber granules and moisture-curable urethane resin.

Various studies have been conducted on synthetic sports surfaces. Benanti et al. [1] performed a force reduction test on seven test pieces and observed that the thickness of the synthetic sports surface significantly affects the shock absorption properties. Andena et al. [2] performed finite element simulations with two athletic tracks and one natural rubber sample to evaluate the effects of surface structure and material properties on the loading rate and energy absorption capacity of the surfaces. Colino et al. [3] evaluated the relationship between two key testing methods—artificial athlete (AA) and advanced artificial athlete (AAA)—for an assessment of the shock absorption and vertical deformation of sports surfaces. They revealed an overall overestimation of shock absorption and underestimation of vertical deformation with the AAA devices and suggested mathematical equations to use AA and AAA devices interchangeably. Tagliabue et al. [4] investigated the effects of environmental variables, such as ultraviolet radiation, the relative humidity, water immersion, and the temperature, using several artificial aging protocols and applied them to prefabricated tracks of different colors and chemical formulations. They found that the combination of accelerated aging protocols and monitoring techniques is a powerful tool for studying the aging of athletic tracks. Kang and Lee [5] adopted three types of ethylene-propylene-diene-methylene (EPDM) rubbers and one type of styrene-butadiene rubber (SBR), and set them up for a 7-day curing process under normal atmospheric conditions after 2 days of curing at a temperature of 30 ± 1 °C and relative humidity of 60% ± 5%. They found that the properties of synthetic sports surfaces using SBR were inferior to those using the three EPDM rubbers, except for shock absorption and vertical deformation. Park [6] conducted tensile tests according to the mixing ratio of EPDM rubber granules and moisture-curable urethane resin, and proposed an appropriate mixing ratio of 20–22%. Hong [7] checked the color difference after an ultraviolet ray-accelerated weathering test with three colors of EPDM granules. Park et al. [8] compared the tensile strength, elongation, and hardness of rubber composites with EPDM granules from recycled car weather-strips and waste tire granules. They confirmed that the tensile strength was unaffected; however, the rubber composites with the EPDM granules exhibited a higher elongation and lower hardness. Park [9] conducted tests on elastic pavement materials using EPDM, urethane, and waste tire granules to evaluate the tensile properties after curing at 23 ± 2 °C for 5 days. The results showed that the elastic pavement with EPDM granules had excellent tensile properties compared with those comprising waste tire granules. The results after curing at moderate (23 ± 2 °C) and high (70 ± 1 °C) temperature ranges showed that the tensile strength increased, while the elongation of the elastic pavement with EPDM granules decreased with an increasing curing temperature. In addition, the test results demonstrated superior tensile properties of the elastic pavement with EPDM granules when cured at a relatively low temperature of −5 ± 2 °C. Choi et al. [10] checked the head injury criterion and slip/skid resistance of a children’s playground using waste polyolefin foam as a buffer. Ko and Ko [11] studied the tensile properties, hardness, rebound elasticity, and tensile strength after aging tests using urethane granules, and presented results on the basic properties of synthetic sports surfaces. They found that synthetic sports surfaces are sensitive to temperature, based on the results that the tensile strength and elongation reduced after the aging test. Choi et al. [12] conducted slip/skid resistance and shock absorption tests with synthetic sports surfaces constituting a sawdust and urethane resin mixture.

Synthetic sports surfaces should provide an appropriate level of safety for athletes with respect to user-to-surface interaction while they perform sports activities [13]. The most important characteristic is the impact of stepping on the ground during exercise. Impact, interpreted as the reaction when a user collides with the ground during walking, running, or jumping, is a widely studied topic in sports research because it is the most likely cause of physical injuries [14]. The resin-bound rubber crumb type is the most widely used in-situ system [1]. In this type of system, the elastic rubber layer is the most influential part of the impact resistance. Polymeric materials are used in the elastic rubber layer to prevent joint and muscle injury because they can reduce the amplitude of incoming shockwaves traveling through the human locomotion system while running or jumping during exercise [1]. In Korea, among numerous polymeric materials, recycled EPDM and recycled SBR are generally used. Recycled EPDM can be obtained from the weather strip of cars, while recycled SBR can be obtained from waste tires. Between the two abovementioned synthetic rubbers, recycled SBR further reduces the magnitude of impact under the same conditions [5]. However, recycled SBR is inferior to EPDM in terms of tensile properties, including the tensile strength and elongation at break [5,8,9]. Although recycled SBR exhibits relatively lower tensile properties than recycled EPDM, it is used by many installers because of its excellent ability to reduce the impact.

Nevertheless, there are limited studies [5,9] on the elastic rubber layer with recycled SBR. Previous studies compared the mechanical and dynamic behavior of elastic rubber layers with SBR and EPDM under the same curing conditions or considered very limited curing conditions, such as only the temperature, as a variable of the testing. Many environmental factors, such as the curing period, temperature, and humidity, can be varied in situ due to seasonal changes, which affect the properties of elastic rubbers and synthetic surfaces. Furthermore, proper proportions of rubber granules and urethane resin, as well as proper construction methods, play an important role in the properties of the elastic rubber layer. However, a considerable number of installers use many kinds of rubber granules and resins with varying mixture proportions, and a variety of construction methods [5]. Therefore, this study investigated the effects of curing conditions, mix proportions, and construction methods on the mechanical and dynamic properties of an elastic rubber layer containing recycled SBR. Furthermore, the results of this test program were compared with the requirements of the current standards for synthetic surfaces to evaluate the applicability of recycled SBR as an ingredient of the elastic rubber layer for synthetic surfaces.

## 2. Experimental Program

### 2.1. Materials

An elastic pavement material, comprising recycled SBR granules and moisture-curable urethane resin, was used for the tests in this study. The recycled SBR granules were produced by Korea E&S (Korea E&S Co., Ltd. Cheonan-si, Chungcheongnam-do, Korea), and their specific gravity was measured to be 1.13 using the specific gravity bottle in KS M 6519 [15]. The grain size of the rubber granules ranged from 1.00 to 3.35 mm. Moisture-curable urethane resin (ELATEC YCB-2009S, Yoogyeong Chemical Co., Ltd. Hwaseong-si, Gyeonggi-do, Korea) was used in this study. The isocyanate group content, specific gravity, percentage volume of nonvolatile matter, and viscosity tested in accordance with KS F 3888-2 [16] were 6.1%, 1.06%, 94.6%, and 4390 mPa·s, respectively.

### 2.2. Test Specimens and Variables

**Figure 2:**
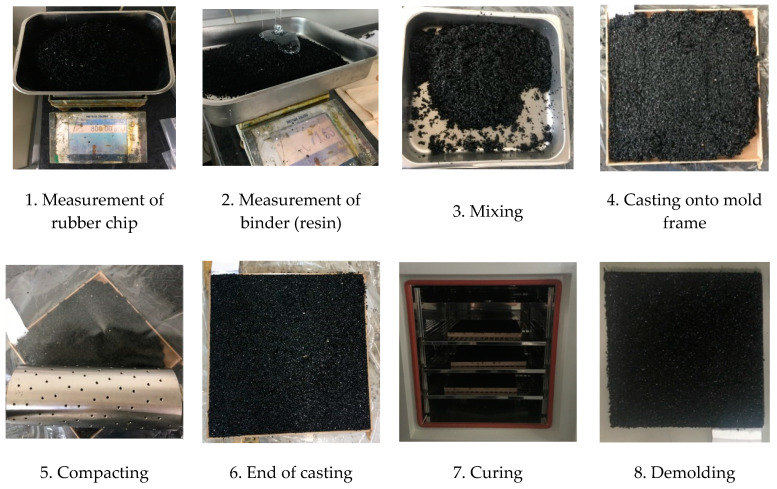
Production process of test samples.

The primary variables of the investigation were the number of compactions, resin–rubber granule ratio, curing period, temperature, and relative humidity. These variables simulate in-situ conditions that may occur during the installation or curing of elastic rubber layers. The size of the mold frame was 300 × 300 × 15 mm^3^, and a Teflon sheet was attached to prevent the test sample from destruction while stripping. Details of the test variables used to evaluate the effects of the number of compactions are listed in Table 1. A tamping roller with a cylindrical shape, a diameter of 100 mm, and a length of 320 mm was used for compaction (see Figure 2). The weight of the tamping roller was 32.1 kg.

The detailed test variables used to evaluate the effects of the curing age are shown in Table 2, while the test variables employed to examine the effects of the resin–rubber granule ratio, temperature, and relative humidity of curing are summarized in Table 3. Each resin (136, 160, 184, and 208 g) was added to 800 g of SBR chips, resulting in resin–rubber granule ratios of 17%, 20%, 23%, and 26%, respectively.

To manufacture the test sample, the recycled SBR chips and resin were measured using an electronic scale. They were then mixed by hand for 2 min and cast onto a mold frame. Later, they were compacted using the tamping roller. After compaction, the test samples were cured under certain conditions in a constant temperature–humidity chamber and then detached from the mold frame, as shown in Figure 2.

### 2.3. Test Setup and Procedure

#### 2.3.1. Direct Tensile Test

**Figure 3:**
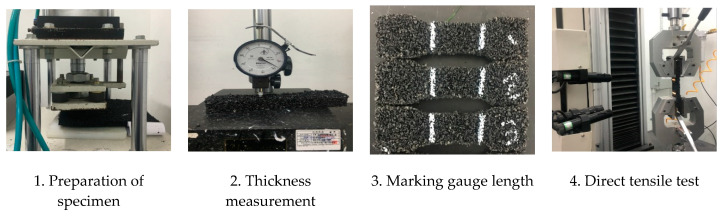
Direct tensile test procedure.

Several test methods have been used to evaluate the tensile properties of synthetic sports surfaces in different countries and associations. World Athletics [17] and European countries evaluate the tensile properties in accordance with the European standard EN 12230 [18]. According to this standard, the width of the specimen is 25 mm and the crosshead speed is 50 mm/min. Meanwhile, the Korean standard KS F 3888-2 [16] defines the width of the specimen as 26 mm and the crosshead speed as 500 mm/min. In this study, the tensile test was carried out in accordance with KS F 3888-2, and the test procedure is shown in Figure 3. Six test specimens were prepared for the direct tensile tests, and a precise dial gauge was used to accurately measure the thickness up to two decimal places. The gauge length for the measurement of elongation was marked using a white marker. The axial load was applied via a 10 kN-capacity universal testing machine (UTM) under displacement control at the loading rate of 500 mm/min. The elongation was measured using a noncontact-type extensometer. The tensile strength and elongation at break were determined by averaging the results from the six samples, calculated using

(1) $$T\left(MPa\right)=\;\frac{F}{W\times t}$$

where *T* is the tensile strength (MPa), *F* is the maximum load (N), *W* is the width of the specimen (mm), and *t* is the thickness of the specimen (mm), and

(2) $$E\left(\%\right)=\;\frac{L}{L_{0}}\times 100$$

where *E* is the elongation at break (%), *L* is the variation in distance between gauge marks at the break point (mm), and *L*_0_ is the gauge length (mm).

#### 2.3.2. Shock Absorption Test

**Figure 4:**
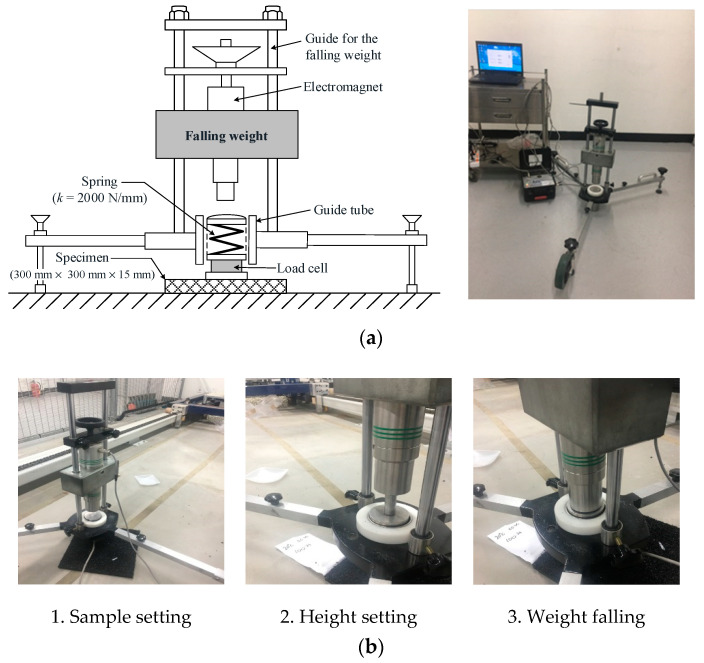
Shock absorption test equipment and procedure: (**a**) Artificial athlete apparatus used for the shock absorption test (impact tester) and (**b**) the shock absorption test procedure.

When testing the dynamic behavior during user–surface interactions, shock absorption and vertical deformation are traditionally considered [13]. Shock absorption, also known as force reduction, is the most significant parameter in synthetic sports surfaces as an injury prevention criterion because it reflects the capability of the surface to reduce impact forces when athletes make contact with the surface [14,19]. In this study, a shock absorption test was carried out in accordance with EN 14808 [20] using AA apparatus (an impact tester), as shown in Figure 4a, and the test procedure is shown in Figure 4b. When a 20 kg weight is dropped from a height of 55 mm, a spring (spring constant of 2000 N/mm) located under the falling weight is impacted. The load cell located under the spring records the maximum peak force (as in Figure 4b). The shock absorption was calculated using the following formula:

(3) $$R\left(\%\right)=\;\left(1-\;\frac{F_{t}}{F_{r}}\right)\times 100$$

where *R* is the force reduction (%), *F_t_* is the measured maximum peak force for the test sample (N), and *F_r_* is the measured maximum peak force for concrete (N).

The procedure was repeated three times, and the mean values of shock absorption from the second and third impacts were recorded. Shock absorption was obtained by averaging the results of the tests on the four points of the specimen.

#### 2.3.3. Vertical Deformation Test

**Figure 5:**
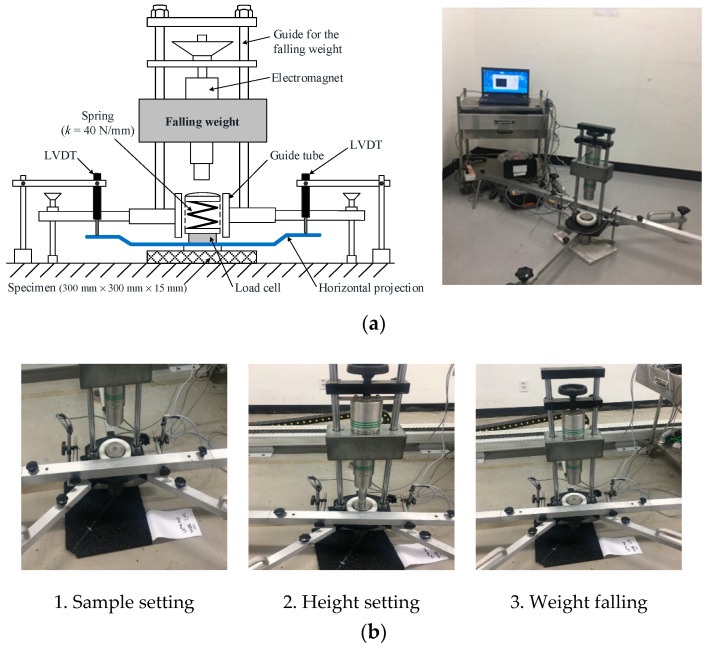
Vertical deformation test equipment and procedure: (**a**) Artificial athlete apparatus used for the vertical deformation test, and (**b**) the vertical deformation test procedure. LVDT: linear variable differential transducer.

The ability to absorb impact forces is influenced not only by the shock absorption capacity, but also by the maximum possible deformation of the surface [21]. Vertical deformation is also a major component of the foot–surface interaction because it expresses the ability of a surface to deform under load [3]. Here, a vertical deformation test was conducted in accordance with EN 14809 [22]. The testing equipment used to measure vertical deformation is similar to the shock absorption test equipment, with only a few parts requiring modification, as shown in Figure 5a. By replacing the spring with a spring constant of 40 N/mm, and by adding a horizontal projection below the load cell and two linear variable differential transducers (LVDTs) to measure vertical deformations, the shock absorption test equipment could be changed into vertical deformation test equipment. As shown in Figure 5b, the spring located under the falling weight is impacted when a 20 kg weight is dropped from the height of 120 mm. The load cell located under the spring records the maximum peak force, and vertical deformation is measured using the two LVDTs simultaneously. Vertical deformation was calculated by the following formula:

(4) $$D\left(mm\right)=\;\left(\frac{1500\;N}{F_{max}}\right)\times f_{max}$$

where *D* is the vertical deformation (%), *f_max_* is the measured maximum deformation of the test sample on the falling weight axis (mm), and *F_max_* is the measured maximum peak force of the test sample (N).

The procedure was repeated three times, and the mean values of deformation from the second and third impacts were recorded. Vertical deformation was obtained by averaging the results of the tests on the four points of the specimen.

## 3. Test Results and Discussion

### 3.1. Effects of the Number of Compactions

**Figure 6:**
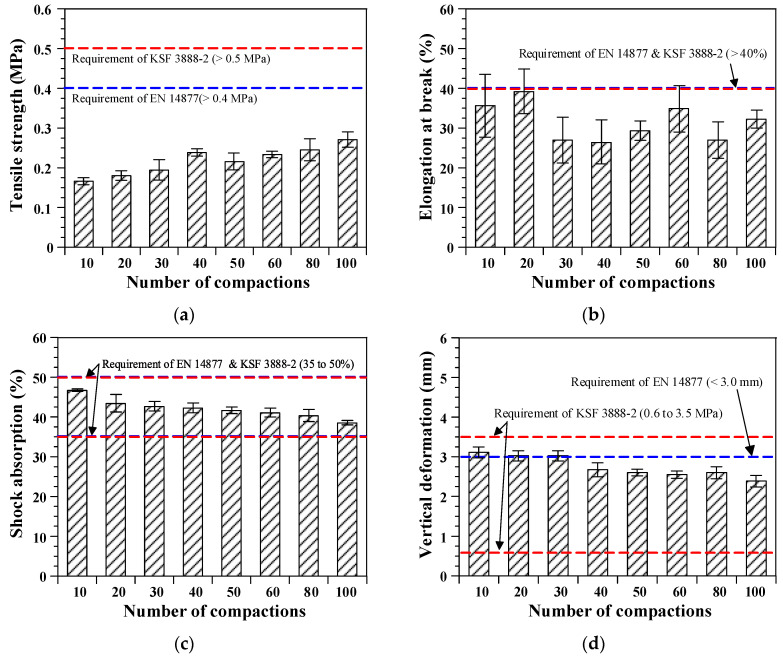
Effects of the number of compactions: (**a**) Tensile strength; (**b**) elongation at break; (**c**) shock absorption; and (**d**) vertical deformation.

Figure 6 shows the results of the measurement according to the number of compactions when casting the elastic rubber layer. As shown in Figure 6a, the tensile strength of the elastic rubber layer improved proportional to the number of compactions. Specifically, the tensile strength increased to 163% when the number of compactions was 100 compared with that when the number of compactions was 10. The increase in tensile strength can be inferred from roller-compacted concrete pavement (RCCP), which is a typical construction method employed for concrete paving. The RCCP method can obtain an excellent strength, owing to the hydration reaction of cement and the effect of internal aggregate interlocking through roller compacting [23]. As a result, the strength of RCCP increases as the compacting energy increases [24]. In the same manner, the tensile strength of the elastic rubber layer also increased owing to the interlocking effect of the rubber granules as the compacting energy increased. Meanwhile, the elongation at break was not affected by the number of compactions. It should be noted that relatively large variations were obtained from the elongation compared to other parameters.

The shock absorption and vertical deformation decreased with an increasing number of compactions (see Figure 6c,d). Shock absorption and vertical deformation decreased by up to 82% and 77%, respectively, when the compaction number was 100 compared with those when the compaction was 10 times. These results were caused by the increased hardness of the elastic rubber layer following roller compaction. Previous studies have shown that the tensile strength and hardness are proportional [9]. Therefore, it is assumed that the elastic rubber layer with increased interlocking of rubber granules exhibits a greater hardness. The shock absorption ability and maximum deformation may be reduced because of hardening of the elastic rubber layer.

The test results of the shock absorption and vertical deformation satisfied the target values specified in both the European Standard (EN 14877 [25]) and Korean Standard (KSF 3888-2 [16]), regardless of the number of compactions. In comparison, the target values of 0.4~0.5 MPa and 40% for the tensile strength and elongation at break, respectively, were not achieved, indicating that the other conditions applied in this test program, i.e., a curing temperature of 20 ± 2 °C, relative humidity of 50% ± 5%, and urethane resin–rubber granule ratio of 23%, were not sufficient to produce satisfactory tensile properties of the elastic rubber layer.

### 3.2. Effect of the Curing Age

**Figure 7:**
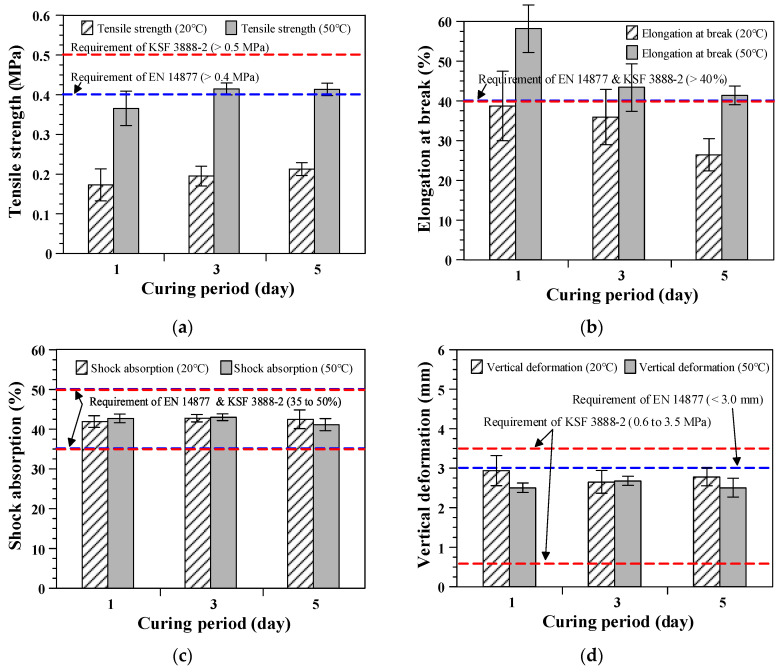
Effects of the curing age: (**a**) Tensile strength; (**b**) elongation at break; (**c**) shock absorption; and (**d**) vertical deformation.

Figure 7 shows the results of the measurements according to the curing age. The tensile strength increased and the elongation at break decreased proportional to the curing age when cured at 20 ± 2 °C. These results are attributed to the progressive hardening of the urethane resin by increasing the curing period. At a relatively high curing temperature of 50 ± 2 °C, the tensile strength increased, but the elongation at break significantly decreased with the increasing curing period from 1 to 3 days. However, a further increase in the tensile strength was not obtained, and the further decrease in the elongation at break was not as significant for a curing age of more than 3 days. These results indicate that high tensile strength results can be obtained at an early age by increasing the curing temperature, because higher temperatures accelerate the hardening and strength development of the urethane resin. Therefore, it is confirmed that the curing period of the elastic rubber layer needs to be adjusted according to the curing temperature.

All specimens exhibited similar shock absorption and vertical deformation, regardless of the curing period and curing temperature, as shown in Figure 7c,d. A previous study [1] indicated that the force reduction ability of sports surfaces is highly dependent on the viscoelastic properties related to the resin–rubber granule ratio and thicknesses of the specimens compared with any other parameter. For this reason, similar shock absorption and vertical deformation results can be obtained for elastic rubber layers cured for different periods and at different temperatures because they have the same thickness and resin–rubber granule ratio.

The elastic rubber layers cured at 50 ± 2 °C for more than 3 days satisfied all requirements of the European Standard EN 14877 [25], including the tensile strength, elongation at break, shock absorption, and vertical deformation. However, the elastic rubber layers achieved an insufficient tensile strength and elongation at break, regardless of the curing period, when they were cured at an ambient temperature of 20 ± 2 °C. Therefore, the curing temperature is a more important factor than the curing period for achieving an appropriate level of mechanical properties of the elastic rubber layers. It can also be concluded that a sufficiently high curing temperature (approximately 50 °C) is required to obtain reasonable tensile properties of elastic rubber layers containing recycled SBR.

### 3.3. Effects of the Curing Temperature and Resin–Rubber Granule Ratio

**Figure 8:**
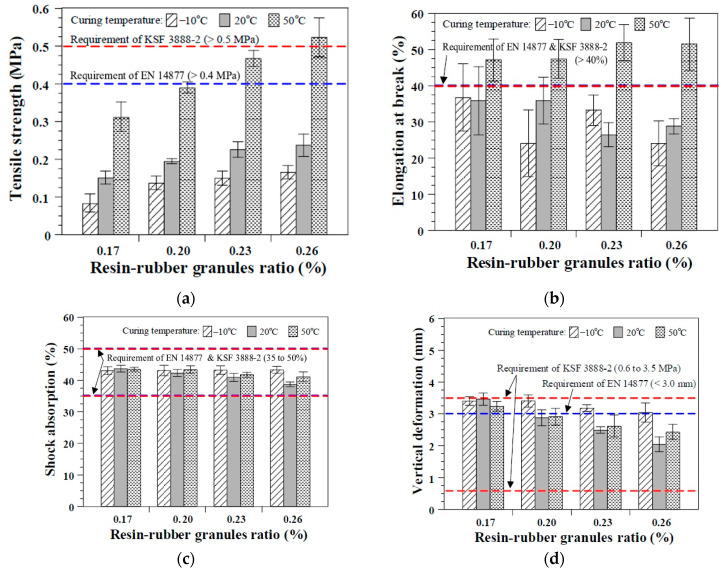
Effects of the curing temperature and resin–rubber granule ratio: (**a**) Tensile strength; (**b**) elongation at break; (**c**) shock absorption; and (**d**) vertical deformation.

Figure 8 shows the results of the measurement according to the curing temperature and resin–rubber granule ratio. In the case of curing at −10 ± 2 °C, the tensile strength decreased to 66% on average, ranging from 55% to 70%, compared with that for curing at 20 ± 2 °C. For curing at 50 ± 2 °C, the tensile strength increased to 210% on average, ranging from 201% to 221%, compared with that for curing at 20 ± 2 °C. In the case of elongation at break (Figure 8b), there was no consistent relationship with the curing temperature for curing at −10 ± 2 °C and 20 ± 2 °C. However, the elongation at break of the elastic rubber layer increased by 32% to 96% when the samples were cured at 50 ± 2 °C compared with those cured at 20 ± 2 °C. Therefore, both the tensile strength and elongation at break improved when increasing the curing temperature from 20 ± 2 °C to 50 ± 2 °C in this study (see Figure 8a,b). These results coincide with the results shown in Figure 7, which exhibits the effects of the curing age on the tensile properties of the elastic rubber layers, which displayed higher tensile properties with an increasing curing temperature, regardless of the curing age. The results of previous research [9] also support the results of this study. In a previous study [9], elastic rubber layers stored in the 70 °C chamber after initial curing exhibited approximately 8% to 50% improvement in the tensile strength owing to the accelerated hardening of the urethane resin. It should be mentioned that wide variations in the elongation at break can be observed in Figure 8b, similar to those that can be observed in Figure 6b and Figure 7b.

**Figure 9:**
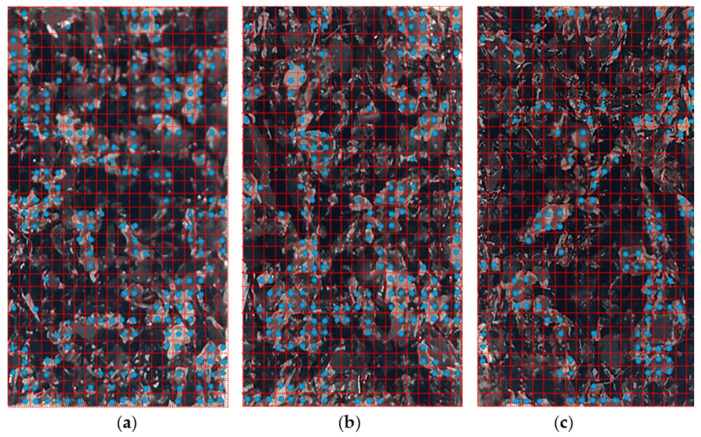
Failure surfaces of elastic rubber layers after the direct tensile test: Curing at (**a**) −10 ± 2 °C, (**b**) 20 ± 2 °C, and (**c**) 50 ± 2 °C.

To provide a better understanding of the test results, an examination of the failure surface was performed after the direct tensile tests in this study. As shown in Figure 9, the failure surfaces were divided into 600 grids to identify the ratio of the white area, considered to have broken in the urethane resin–rubber granule interfaces, and the black area, considered to have broken within rubber granules. The ratios of the area of failure in the urethane resin–rubber granule interfaces to the gross area of the elastic rubber layer specimens were approximately 34%, 30%, and 21% for curing temperatures of −10, 20, and 50 °C, respectively, indicating that more failures occurred at the interfaces between the urethane resin and rubber granules with a decreasing curing temperature. Therefore, the increased tensile properties of the elastic rubber layers at higher temperatures can be attributed to the improved binding force of the urethane resin causing fractures in the rubber chips, rather than the resin–rubber granule interfaces.

The effects of the curing temperature on the shock absorption and vertical deformation of the elastic rubber layer are not significant, as shown in Figure 8c,d. This observation coincides with the results of Park [9], which showed that shock absorption and vertical deformation are not affected by the curing temperature.

Figure 8 also displays the effects of the resin–rubber granule ratio on the mechanical properties of the elastic rubber layer. As shown in Figure 8a, the tensile strength improved as the quantity of resin increased (as the resin–rubber granule ratio increased), regardless of the curing temperature. This result is consistent with the results from Park [9]. They tested elastic rubber layers containing several types of rubber chips with varying mixture proportions, and demonstrated enhanced tensile strengths of elastic rubber layers by increasing the urethane binder content for mixing. It was explained that this improvement is a result of the increased number of bonds between the rubber granules provided by the higher amount of resin coated on the rubber granule surface. Meanwhile, there was no significant benefit for the elongation at break at higher resin–rubber granule ratios (see Figure 8b).

Shock absorption and vertical deformation of the elastic rubber layer decreased with an increasing resin–rubber granule ratio, except for the specimens cured at −10 °C. In particular, all of the specimens with different resin–rubber granule ratios exhibited quite similar shock absorption values and slightly decreased vertical deformation with an increasing resin–rubber granule ratio for curing at −10 °C. This result is attributed to the insufficient curing with a slower rate and freezing effects of the elastic rubber layer due to the very low temperature, which can result in a quite similar impact resistance. Both the shock absorption and vertical deformation decreased with an increasing resin–rubber granule ratio for curing at 20 and 50 °C owing to the increase in the hardness of the elastic rubber layers with a higher amount of resin. In summary, increasing the amount of resin in the elastic rubber layer mix is beneficial for the tensile strength, while it decreases shock absorption and vertical deformation.

Most of the specimens shown in Figure 8 satisfied the shock absorption and vertical deformation requirements specified in both Korean Standard KSF 3888-2 [16] and European Standard EN 14877 [25]. The elastic rubber layers with the resin–rubber granule ratio of 0.17 or cured at the temperature of −10 °C exhibited slightly higher vertical deformations than the specified values in EN 14877 [25]. Nevertheless, these values are within the acceptance criteria specified in KSF 3888-2 [16]. Only the elastic rubber layers cured at the high temperature of 50 °C could satisfy the target values for the tensile strength and elongation at break specified in EN 14877 [25]. These results also support the aforementioned conclusions that the curing temperature is a more important factor than other parameters, and a sufficiently high curing temperature (approximately 50 °C) is required to obtain reasonable tensile properties of the elastic rubber layer containing recycled SBR.

### 3.4. Effects of the Relative Humidity and Resin–Rubber Granule Ratio

**Figure 10:**
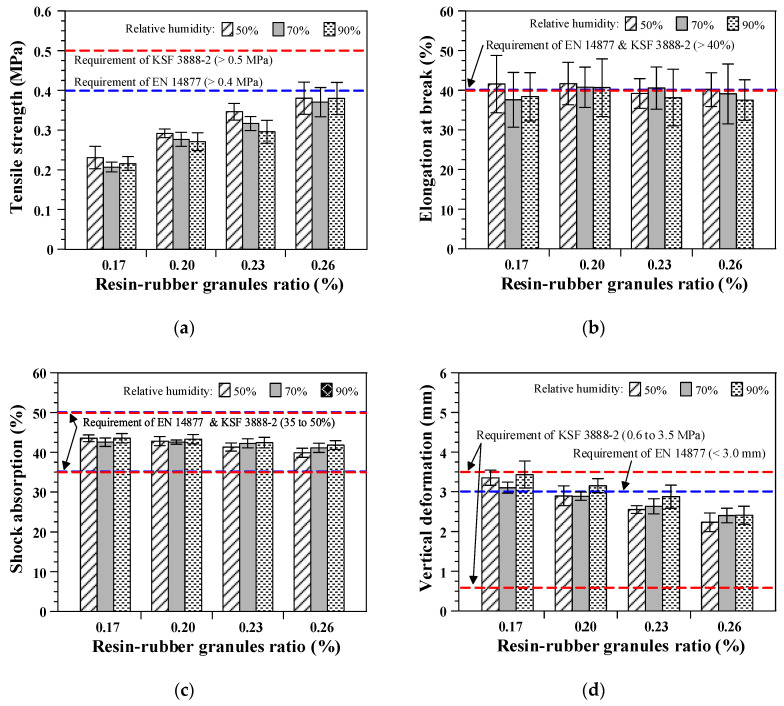
Effects of the relative humidity and resin–rubber granule ratio: (**a**) Tensile strength; (**b**) elongation at break; (**c**) shock absorption; and (**d**) vertical deformation.

Figure 10 presents the results of the measurement according to the relative humidity conditions for curing and resin–rubber granule ratio. The elastic rubber layer specimens cured under different humidity conditions attained almost the same tensile strength, elongation at break, shock absorption, and vertical deformation values. Polyurethane resin is a polymeric material with urethane linkage (–NHCOO–), which is formed by the polyaddition reaction of isocyanate (–NCO) with polyol (–OH) [26,27]. Sufficient humidity conditions need to be maintained for the curing of urethane resin because polyol for the polyaddition reaction is generated from moisture in the air. Furthermore, the amount of polyol consumed by the polyaddition reaction is determined by the isocyanate content of the urethane resin. The recommended relative humidity for the polyaddition reaction of urethane resin used in this study is approximately 40% [28]. Therefore, the relative humidity conditions in Figure 10, ranging from 50% to 90%, can be considered sufficient for providing the polyol required in the polyaddition reaction with isocyanate. As a result, quite similar test results were obtained to those displayed in Figure 10, although varying humidity conditions were applied for curing.

Under the same humidity conditions, improved tensile strength and decreased shock absorption and vertical deformation results were obtained by increasing the resin–rubber granule ratio. The results of elongation at break were inconsistent with the amount of resin in the mix. These results agree with those obtained from the tests shown in Figure 8, and were used to investigate the effects of the curing temperature and resin–rubber granule ratio. The improvement in the tensile strength is attributed to the better adhesion between each rubber granule provided by the sufficient amount of resin coated on the rubber granules. In contrast, a higher amount of urethane resin increases the hardness of the elastic rubber layers, resulting in decreased vertical deformation and shock absorption.

Similar to the results obtained from the tests shown in Figure 8, elastic rubber layers with SBR with varying resin–rubber granule ratios and cured under different humidity conditions could reasonably satisfy the performance criteria specified in the standards, except for the tensile strength. Although a higher amount of resin increases the tensile strength of the elastic rubber layers, the target values specified in both EN 14877 [25] and KSF 3888-2 [16] could not be achieved. This result, compared with those shown in Figure 8, demonstrates the importance of a sufficiently high curing temperature for ensuring an appropriate level of tensile properties of the elastic rubber layer over the requirements of the standards.

## 4. Conclusions

In this study, the mechanical properties of an elastic rubber layer produced using recycled SBR granules were evaluated with respect to the curing conditions. Therefore, within the scope of this study, the following conclusions can be drawn:

(1) The tensile strength increased to 163% when the number of compactions was 100, compared with that when the number of compaction cycles was 10. However, shock absorption and vertical deformation decreased by 82% and 77%, respectively, when the compaction number was 100 compared with those when the number of compactions was 10;
(2) The tensile strength increased, whereas the elongation at break decreased with an increasing curing period owing to progressive hardening of the urethane resin. Furthermore, a higher curing temperature accelerated the hardening and strength development of urethane resin at an early age. The shock absorption and vertical deformation remained unchanged, regardless of the curing age;
(3) The tensile properties, including both the tensile strength and elongation at break, significantly increased with increasing the curing temperature from 20 to 50 °C. However, the effects of the curing temperature on the shock absorption and vertical deformation of the elastic rubber layers were not significant;
(4) The tensile strength improved, but the vertical deformation decreased, as the resin–rubber granule ratio increased. The elongation at break and shock absorption were not significantly affected by the amount of resin used for mixing;
(5) The effects of the relative humidity conditions on the mechanical and dynamic behaviors of the elastic rubber layer with recycled SBR granules were not significant. A relative humidity of over 50% is sufficient for providing the moisture required for hardening the urethane binder;
(6) The curing temperature has a more pronounced effect on the mechanical properties of the elastic rubber layer with recycled SBR granules than other parameters related to the making or curing of the elastic rubber layer;
(7) Elastic rubber layers with SBR only satisfied the performance requirements specified in the standards for resin–rubber granule ratios above 23% and curing at 50 °C. Therefore, a sufficient amount of resin with a sufficiently high curing temperature is required to obtain reasonable tensile properties of the elastic rubber layer containing recycled SBR.

## Figures and Tables

**Table 1:** Test variables used to evaluate the effects of the number of compactions.

Specimen	Temperature (°C)	Relative Humidity (%)	Rubber Chip Weight (g)	Resin Weight (g)	Curing Period (Days)	Number of Compactions
20-50-10T	20 ± 2	50 ± 5	800	184	7	10
20-50-20T						20
20-50-30T						30
20-50-40T						40
20-50-50T						50
20-50-60T						60
20-50-80T						80
20-50-100T						100

**Table 2:** Test variables employed to evaluate the effects of the curing period.

Specimen	Temperature (°C)	Relative Humidity (%)	Rubber Chip Weight (g)	Resin Weight (g)	Curing Period (Days)	Number of Compactions
20-50-1D	20 ± 2	50 ± 5	800	184	1	40
20-50-3D					3	
20-50-5D					5	
50-50-1D	50 ± 2				1	
50-50-3D					3	
50-50-5D					5	

**Table 3:** Test variables applied to evaluate the effects of the mixing ratio, temperature, and humidity.

Specimen	Temperature (°C)	Relative Humidity (%)	Rubber Chip Weight (g)	Resin Weight (g)	Curing Period (Days)	Number of Compactions
−10-0-17B	−10 ± 2	-	800	136	7	40
−10-0-20B				160		
−10-0-23B				184		
−10-0-26B				208		
20-50-17B	20 ± 2	50 ± 5		136		
20-50-20B				160		
20-50-23B				184		
20-50-26B				208		
20-70-17B		70 ± 5		136		
20-70-20B				160		
20-70-23B				184		
20-70-26B				208		
20-90-17B		90 ± 5		136		
20-90-20B				160		
20-90-23B				184		
20-90-26B				208		
50-50-17B	50 ± 2	50 ± 5		136		
50-50-20B				160		
50-50-23B				184		
50-50-26B				208		
